# Perovskite nanocomposites: synthesis, properties, and applications from renewable energy to optoelectronics

**DOI:** 10.1186/s40580-024-00440-7

**Published:** 2024-09-09

**Authors:** Yunseok Choi, Sangmoon Han, Bo-In Park, Zhihao Xu, Qingge Huang, Sanggeun Bae, Justin S. Kim, Sun Ok Kim, Yuan Meng, Seung‐Il Kim, Ji‐Yun Moon, Ilpyo Roh, Ji-Won Park, Sang‑Hoon Bae

**Affiliations:** 1 Department of Mechanical Engineering and Materials Science, Washington University in St. Louis, St. Louis, MO 63130 USA; 2 Department of Mechanical Engineering, Massachusetts Institute of Technology, Cambridge, MA 02139 USA; 3 Research Laboratory of Electronics, Massachusetts Institute of Technology, Cambridge, MA 02139 USA; 4 The Institution of Materials Science and Engineering, Washington University in St. Louis, Saint Louis, MO 63130 USA; 5 Department of Energy Systems Research and Department of Materials Science and Engineering, Ajou University, Suwon, 16499 South Korea; 6 R&D CENTER, M.O.P Co., Ltd, Seoul, 07281 South Korea; 7 R&D Center of JB Lab Corporation, Gwanak-Gu, Seoul, 08788 Republic of Korea

**Keywords:** Perovskite, Nanocomposite, Heterostructure

## Abstract

The oxide and halide perovskite materials with a ABX_3_ structure exhibit a number of excellent properties, including a high dielectric constant, electrochemical properties, a wide band gap, and a large absorption coefficient. These properties have led to a range of applications, including renewable energy and optoelectronics, where high-performance catalysts are needed. However, it is difficult for a single structure of perovskite alone to simultaneously fulfill the diverse needs of multiple applications, such as high performance and good stability at the same time. Consequently, perovskite nanocomposites have been developed to address the current limitations and enhance their functionality by combining perovskite with two or more materials to create complementary materials. This review paper categorizes perovskite nanocomposites according to their structural composition and outlines their synthesis methodologies, as well as their applications in various fields. These include fuel cells, electrochemical water splitting, CO_2_ mitigation, supercapacitors, and optoelectronic devices. Additionally, the review presents a summary of their research status, practical challenges, and future prospects in the fields of renewable energy and electronics.

**Graphical abstract:**

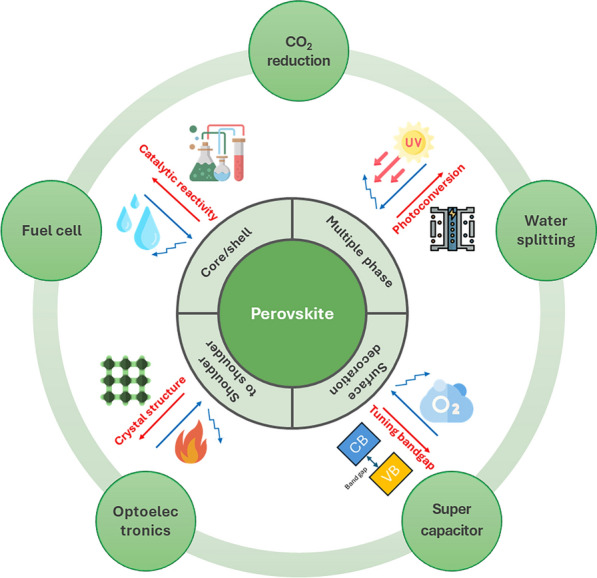

## Introduction

The perovskite structure, which is constructed with an ABX_3_ atomic arrangement, has attracted increasing attention due to its ability to be implemented in various research areas. This is achieved by manipulating the elements of A, B, and X. The unit cell is formed by A cation groups and BX_6_ octahedra sharing corners. Distortion of the unit cell typically occurs due to the rotation of BX_6_, which alters the B-X, B-X-B angles, leading to changes in the optical, electronic, and band structure. Depending on the X atom, perovskite materials have been classified as oxide perovskite (oxygen) and halide perovskite (Cl, Br, I), each offering distinctive material properties [1]. The perovskites have been recognized for their outstanding performance in a range of applications, including supercapacitors, fuel cells, water splitting, solar cells, photocatalysts, CO_2_ reduction, and so on [2–13]. 

Although these oxide and halide perovskites offer their unique material properties in many applications, they also have limitations and numerous research efforts have been made to overcome these limitations. Oxide perovskites suffer from low photoconversion efficiency and low catalytic reactivity due to the low carrier density in the materials, which hinders efficient charge transport. In contrast, halide perovskites have low chemical stability and reliability in light, heat and moisture [14–24]. Efforts to address these shortcomings of perovskites have included tailoring morphology and texture properties, partial substitution of AB cations and X ions, and fabrication of heterogeneous interfaces. Nevertheless, single-phase perovskites continue to face challenges, necessitating innovative approaches. It has motivated the development of nanocomposites to meet diverse performance requirements simultaneously [25–35]. The materials for nanocomposite formation include perovskite materials [36–56], metal oxides (e.g., Si, Ti, Zr) [57–66], sulfide materials (e.g., CdS, PbS, ZnS) [67, 68], polymers [69, 70], and glass [71]. The nanocomposite formation assists in optimizing the crystal structure, enhancing structural stability, and providing additional pathways for charge transport in perovskites. Thus, it has been regarded as a promising approach to compensate perovskite properties. This review aims to comprehensively summarize recent advancements in perovskite nanocomposites, focusing on their synthesis methodologies and applications in electrochemical and optoelectronic fields. The synthesis methodologies of perovskite nanocomposites can be classified into two principal categories: in-situ and post-synthesis techniques. This review also examines the latest research trends, practical challenges, and emerging opportunities associated with these nanocomposites, particularly in applications such as fuel cells, electrochemical water splitting, electrochemical CO_2_ reduction, supercapacitors, LEDs, and solar cells. It provides insights into the potential and limitations of perovskite nanocomposites in enhancing the performance and stability.

## Perovskite nanocomposites (materials property)

**Fig. 1:**
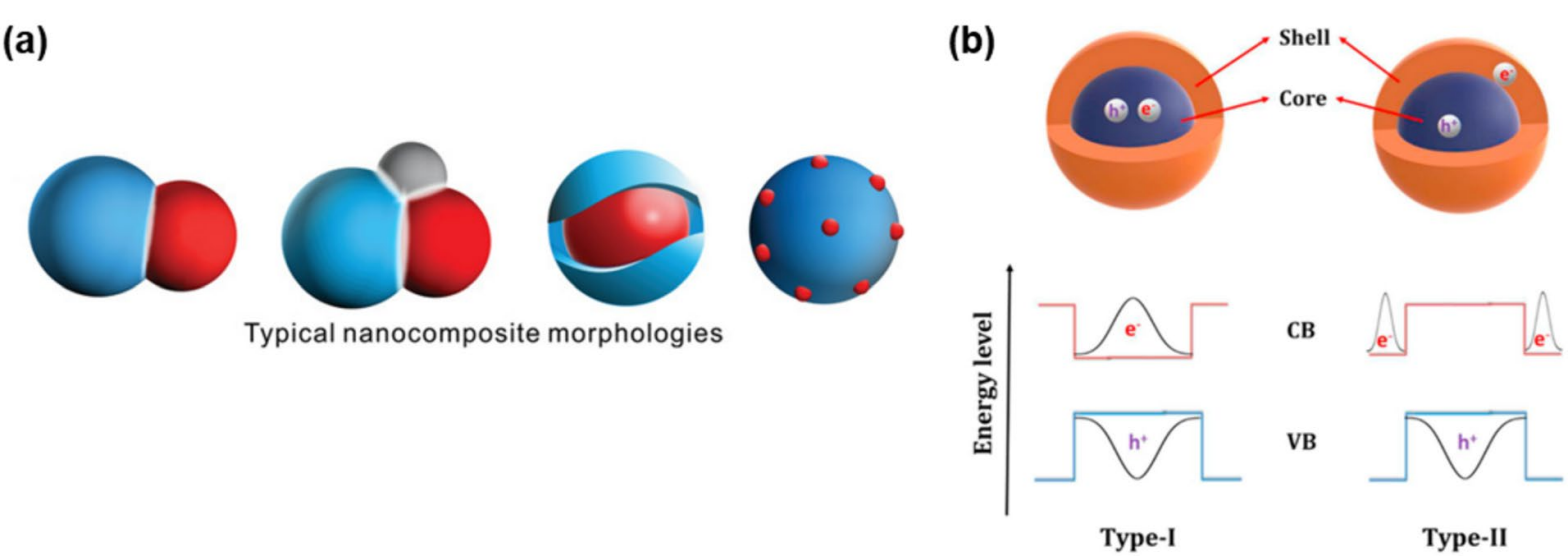
Schematics of various perovskite nanocomposites. **a**. From left to right, the typical structures of nanocomposites are dual nanocomposite, triple nanocomposite, core/shell nanocomposite, and socketed nanocomposite (surface decoration) [182]. **b**. Energy level diagram as Type I and Type II of core/shell structure [183]

Nanocomposite structures exhibit a variety of forms, including non-core/shell bonded structures, and core/shell structures. Non-core/shell nanocomposites are typically synthesized from a single precursor solution and consist of different crystal structures and compositions. These unique compositions often enhance catalytic performance due to the formation of unique and close interfaces between different heterostructures. Hybrid nanocomposites composed of different compositions of perovskite materials or different materials are synthesized, creating intimate connections between phases by using one mother precursor solution to synthesize multiple phases with different proportions [55, 57, 72]. The synthesized nanocomposites, comprising small units, prioritize interface formation throughout manufacturing processes, yielding nanoscale products abundant in heterointerfaces. It frequently facilitates the optimization of perovskite’s electronic structure and accelerates the diffusion paths of ions and electrons by fostering interfaces between diverse crystal phases within nanocomposites. Thus, the formation of interfaces between different crystal phases of nanocomposites can induce unexpected physical and chemical properties, and strong interactions can promote the long-term stability of the synthesized nanocomposites [56, 61, 73, 74]. These structural advantages demonstrate significant improvements in tuned bandgap for catalytic activity, electron mobility for electrical conductivity, magnetic properties, energy capacity and density, surface area and structure, and long-term stability in perovskite oxide-based nanocomposites. For uniform mixing, spray pyrolysis is used to synthesize nanocomposites with high surface area and uniform structure [62–64]. Furthermore, hybrid nanocomposites comprising two or more perovskite types with disparate crystal structures or compositions can interact closely through shoulder-to-shoulder contact [57, 62] or surface decoration patterns (Fig. 1a) [75–77]. A simple method to obtain tightly interconnected nanocomposites is to mix precursors of different components and then proceed with simultaneous crystallization to form the composite. However, this typical route may lack control over size and microstructure. Small amounts of perovskite can be added to a perovskite, often in the form of surface decoration. Surface decoration of perovskite based oxides has proven to be a unique method to enhance catalytic performance due to the formation of highly efficient interfaces.

Within these composite structures, core/shell structures are widely favored for their ability to combine the strengths of each constituent material (Fig. 1a). They offer the added benefit of enhancing the stability and performance of halide perovskite [78–81]. The heterostructure can exhibit either epitaxial or non-epitaxial relationships, depending on the properties of the materials and the chemical approaches used in their preparation. For epitaxial structure, the distribution of electron/hole wave functions within each component is crucial in determining the composite structure. The energy levels of the valence band (VB) and conduction band (CB) of two semiconductors determine the physicochemical properties of the core/shell nanostructure. When the shell materials have a wider energy bandgap than the core materials, and the conduction band and the valence band edges of the core materials are present in a region smaller than the energy gap of the shell, this leads to the confinement of electrons and holes in the core. This confinement results in the formation of a Type I band structure (Fig. 1b). This nanocomposite structures have been applied to the optoelectronic field of perovskite with the objective of improving its optical properties and increasing its stability [23, 24, 48, 82, 83]. The wide bandgap of the shell materials traps the carriers in the core materials, thereby passivating defects on the core surface. This reduces the impact of defects on the surface on the reaction of the core, resulting in an increase in photoluminescence quantum yield (PLQY). A Type II band structure forms when the conduction band and valence band edge of the core materials are lower or higher than the shell materials. In this case, one type of carriers (either electron or hole) is mainly confined to the core side, while the other type of carriers is stored in the shell side. The Type II band alignment is expected to have fundamentally different properties mainly due to the spatial separation of carriers [84–86]. In the context of optical materials, such heterostructures can effectively confine or transfer carriers generated from one side to the other. Consequently, these nanomaterials are employed in a multitude of applications, including the enhancement of light emission, the optimization of solar cell efficiency, and the augmentation of catalytic activity [48, 84, 85, 87–94].

## Synthesis of perovskite nanocomposites

**Fig. 2:**
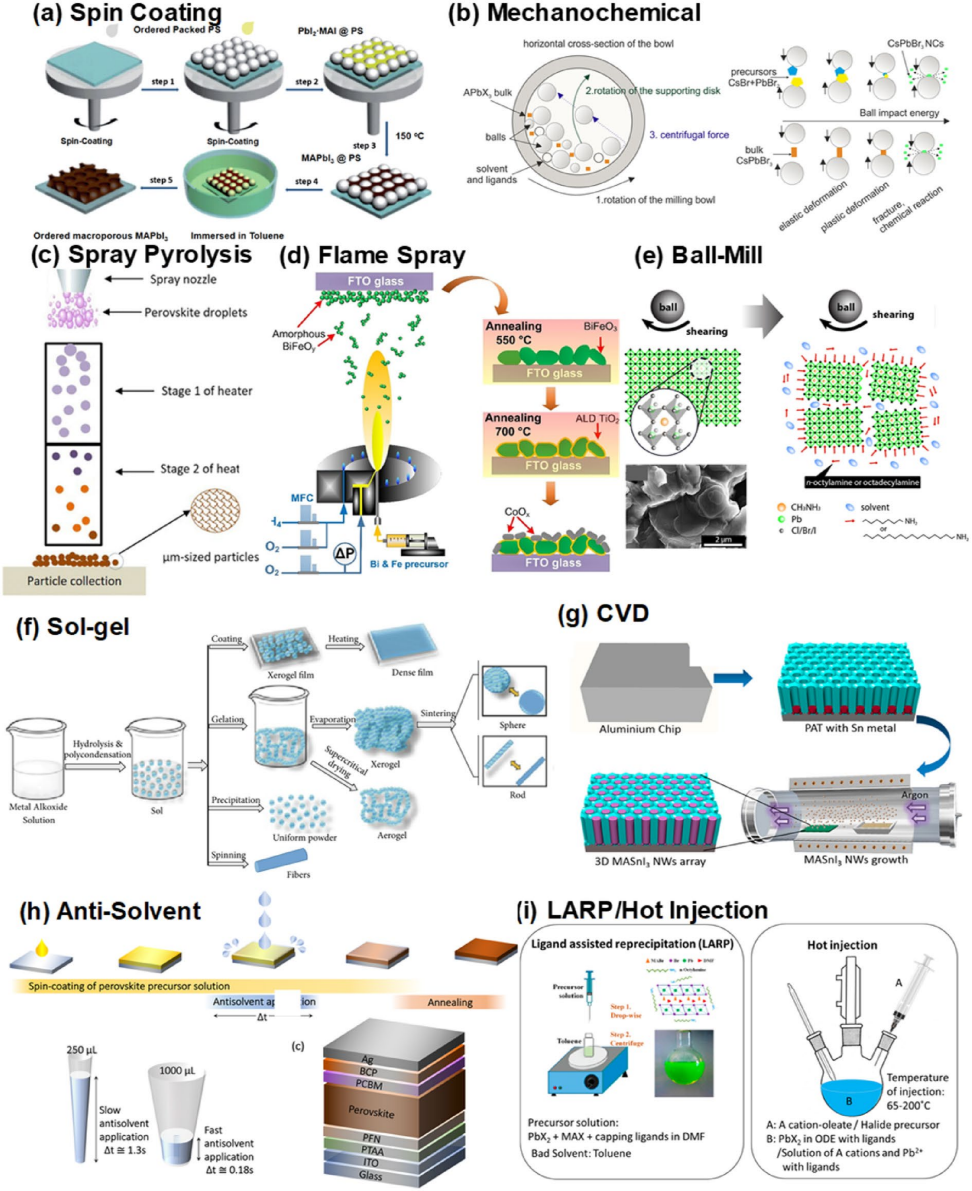
Various synthesis methods for perovskite and nanocomposite structures. **a**. Spin coating [184]. **b**. Mechanochemical [96]. **c**. Spray pyrolysis [185]. **d**. Flame spray pyrolysis [186]. **e**. Ball mill [95]. **f**. Sol–gel [187]. **g**. CVD [188]. **h**. Anti solvent [191]. **i**. LARP [189] and Hot injection [190]

The synthesis of perovskite nanocrystal (NC)s can be broadly divided into two categories: top-down and bottom-up approaches. Top-down approaches involve reducing and breaking down macroscopic materials into nanostructures and particles, which can be achieved through mechanical mills (Fig. 2b), ball-mill (Fig. 2e), and chemical methods [95, 96]. Bottom-up methods involve building structures from ions, molecules, and clusters in liquid and vapor phases. These include sol–gel (Fig. 2f) [97–104], hydrothermal [105–109], high-temperature injection (Fig. 2i) [110–112], ligand assisted reprecipitation (LARP) (Fig. 2i) [113, 114], solid-state reactions [115–122], (flame) spray pyrolysis (Fig. 2c,d) [117, 123–125], chemical vapor deposition (CVD) (Fig. 2g) [126], and spin coating (Fig. 2a) [127–130]. Additionally, perovskite nanocomposites are composites of two or more materials, which allows for the distinction between two scenarios: synthesis of the two or more materials simultaneously (in-situ) or separate post-processing of each material. As previously mentioned, there are several types of perovskite nanocomposites, including core/shell and non-core/shell.

### Core/shell structure

Core shell formation strategies include surface overgrowth of shell materials, multiple shell coatings, and embedding perovskite NCs in the shell matrix [131, 132]. Epitaxial growth is a representative synthesis strategy that involves the direct introduction of a shell precursor into the solution immediately after the core NC is formed. The main process is the binding of ligands to the NC surface, which facilitates the deposition of the shell material onto the core and enables its dispersion in solution without aggregation. The ligands are released to allow for the growth of new atoms, which subsequently re-bind to the surface, thereby initiating a repeating cycle that results in the formation of the core/shell material. Surface passivation, which is applied through the epitaxial overgrowth, has been widely applied in various semiconductors, including metal oxide and metal sulfide, due to its advantages of eliminating trap states, increasing stability, and enhancing photophysical properties. In addition, the formation of the core/shell structure in this manner results in lattice strain due to lattice mismatch, which is related to the diameter of the core and the thickness of the shell [133]. The Matthews-Blakeslee theory is applicable to this phenomenon, and the critical shell thickness that can be grown without causing lattice strain can be obtained. The relationship between critical thickness, lattice mismatch, and dislocation formation demonstrates that as the size of the core decreases, the energy required to compress it increases, resulting in a thicker shell.

**Fig. 3:**
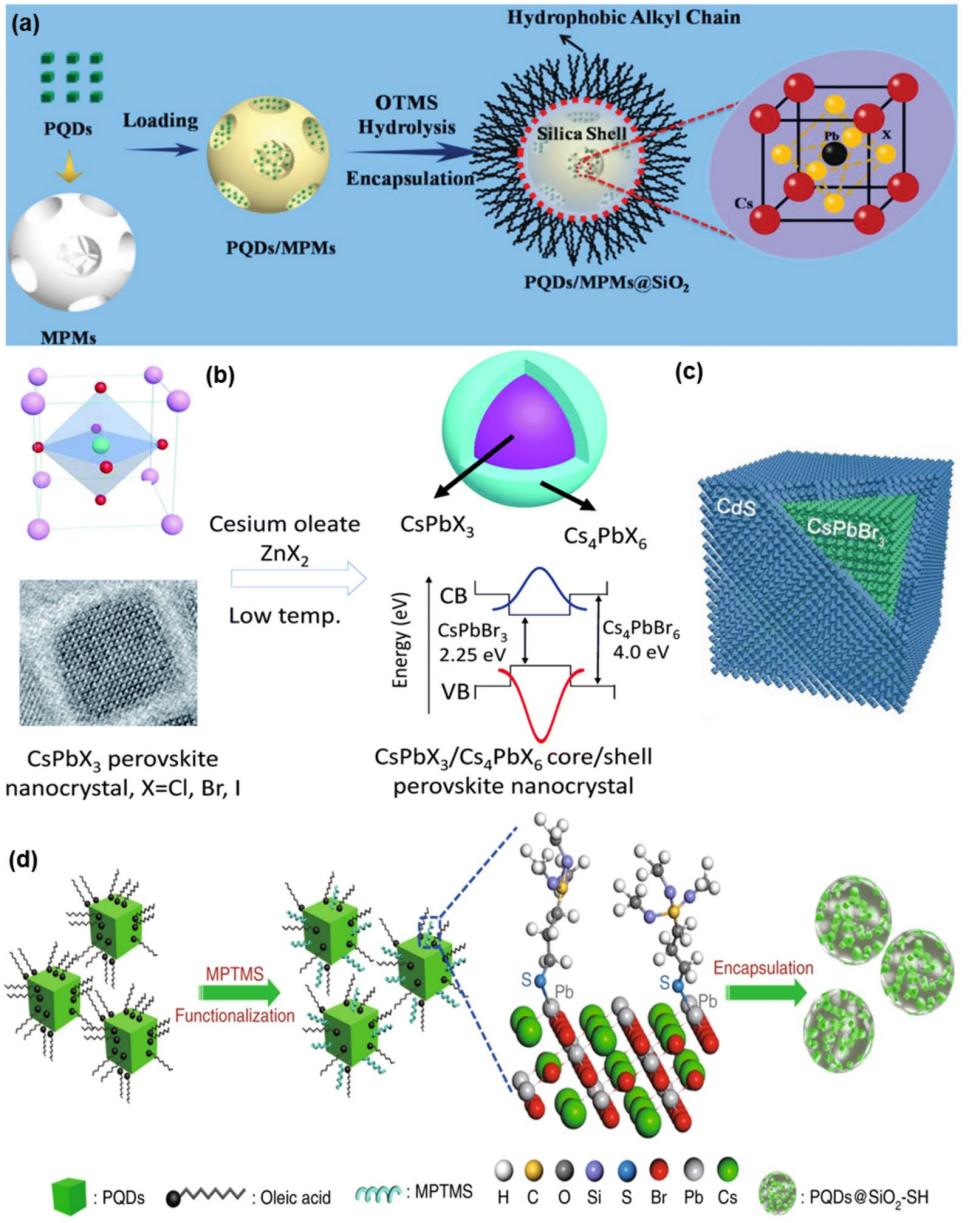
Synthesis methods for core/shell perovskite nanocomposite fabrication (In-situ, and post synthesis) **a**. CsPbBr_3_‐Quantum dots-polystyrene-silica hybrid microsphere structures with significantly improved stability for white LEDs [80]. **b**. Formation CsPbBr_3_-Cs_4_PbBr_6_ core/shell NCs [78]. **c**. Single halide perovskite-semiconductor core/shell quantum dots [192]. **d**. Illustration of Pb–S bonding-based perovskite-silica nanodots [134]

A variety of synthesis methods, including ion exchange, hot injection, spin coating, LARP, wet chemical, sol–gel, and others, have been employed for this synthesis, either in situ or post-synthesis, depending on the specific objective and the material. Jia et al. synthesized CsPbX_3_-Cs_4_PbX_6_ core/shell nanocrystals by in-situ process using hot-injection method (Fig. 3b) [78]. This research presents a technical approach and optimized process conditions for synthesizing nanocrystals with high photoluminescent efficiency, enhancing their potential applications in optoelectronic devices. Green-light-emitting CsPbBr_3_ nanocrystals are prepared as seeds using a high-temperature hot injection method at temperatures above 170 ℃. This process involves the use of cesium oleate and zinc bromide. Afterwards, the shell coating process involves the rapid injection of additional halogen sources into the CsPbBr_3_ nanocrystal seeds. This is done under optimized conditions that facilitate the formation of the hexagonal Cs_4_PbBr_6_ phase. As another example of in-situ synthesis, Tang et al*.* synthesized CsPbBr_3_-CdS core/shell quantum dots (QDs) (Fig. 3c) [192]. To fabricate the CsPbBr_3_ core, Cs-oleate is prepared using Cs_2_CO_3_, 1-octadecene (ODE), and oleic acid (OA), which is then reacted with PbBr_2_ to form QDs. Then, to grow the CdS shell on the CsPbBr_3_ core, Cd-oleate and sulfur are used as shell precursors. These materials are added to the CsPbBr_3_ reaction mixture and reacted at appropriate temperatures to complete the core/shell structure. This approach enables the formation of a stable and efficient core/shell structure, enhancing the quantum dot's stability and optical properties.

As an example of post-synthesis method, Yang et al*.* synthesized cesium lead bromide (CsPbBr_3_) perovskite quantum dots (PQDs) within mesoporous polystyrene microspheres (MPMs), subsequently coated with a silica shell by hydrolysis and encapsulation process, and the stable hybrid microspheres are used in the development of white light-emitting diodes (LEDs) (Fig. 3a) [80]. The PQDs are first embedded into the MPMs. This is followed by a hydrolysis process to form a silica coating over the MPMs, effectively encapsulating the PQDs. This encapsulation strategy protects the PQDs from direct environmental exposure, thereby improving their stability. The silica shells prevent direct contact between the PQDs and external erosive agents such as water and solvents, providing significant resistance to degradation. This encapsulation is demonstrated to maintain substantial fluorescence intensity even after 30 days of exposure to challenging conditions, surpassing the stability metrics of previously reported systems. Li et al. developed a Pb–S bonding-based method to synthesize perovskite-silica nanodots which enhances water resistance of the perovskite nanodots (Fig. 3d) [134]. Pre-synthesized CsPbBr_3_ nanodots were functionalized with (3-mercaptopropyl) trimethoxysilane (MPTPMS) which formed Pb–S bonding with the perovskite via hydrolysis and condensation. The perovskite nanodots are encapsulated by silica, and that kept their emission for six weeks in water.

### Non-core/shell structure

**Fig. 4:**
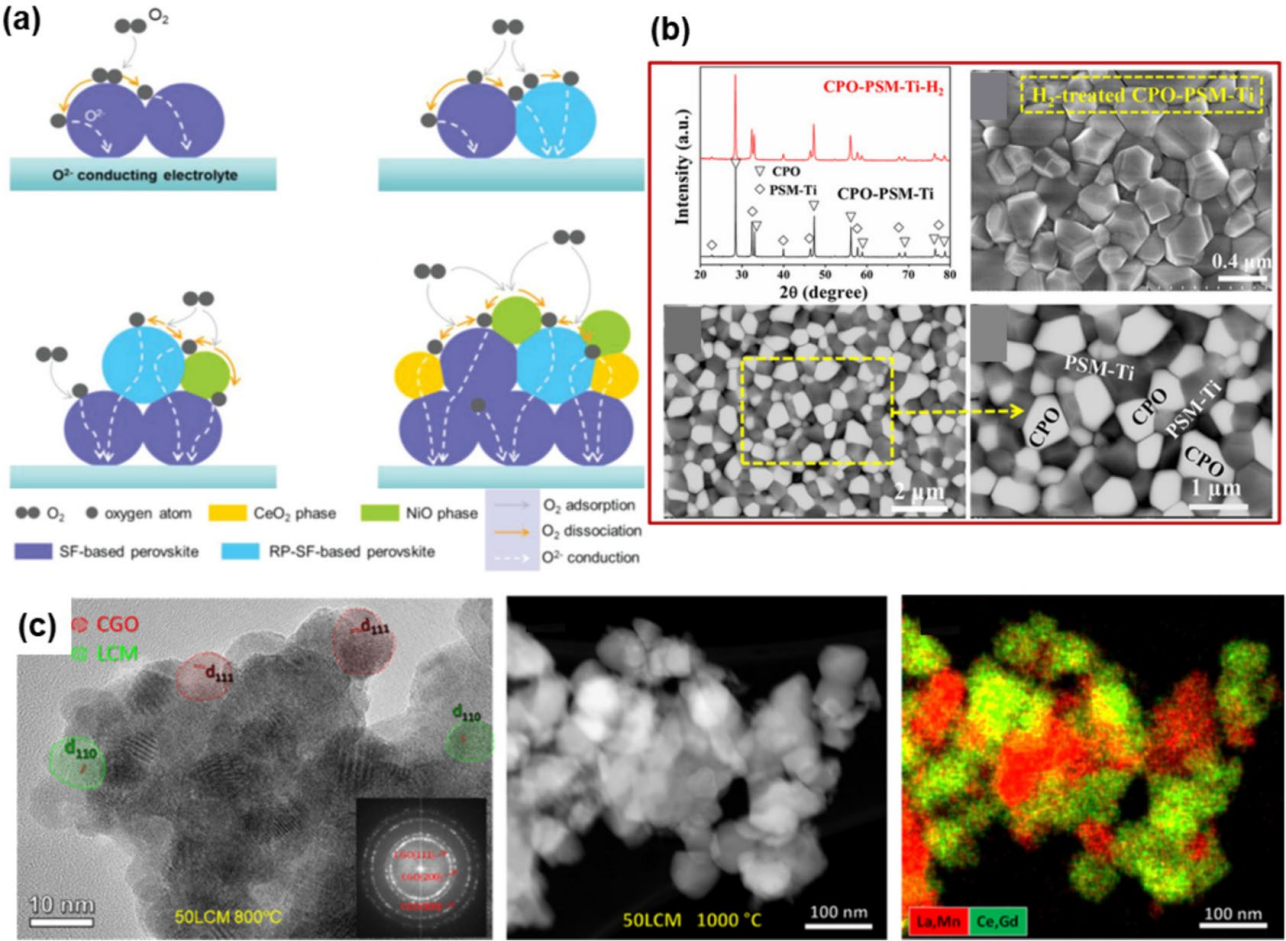
Various Non-core/shell structures: multi-phase and shoulder to shoulder **a**. A cobalt‐dree multi‐phase nanocomposite as near‐ideal cathode of SOFC [135]. **b**. Hydrogen purification through a highly stable dual‐phase oxygen‐permeable membrane [136]. **c**. La_0.98_Cr_0.75_Mn_0.25_O_3-δ_-Ce_0.9_Gd_0.1_O_1.95_ (LCM-CGO) nanocomposite layers by the Spray pyrolysis method [62]

First, nanoscale hybrid composites commonly employ a one-pot mother precursor. This approach leads to the simultaneous crystallization of stable thermal formulas, resulting in the formation of twin or multiple perovskite phases. By employing sol–gel or hot injection synthesis, researchers can produce two or more nanocomposites. These multiphase structures are anticipated to exhibit excellent catalytic performance due to synergistic effects resulting from the unique and intimate interfaces between different heterostructures or due to the structural segregation caused by dopants, resulting in nanocomposites with distinct structures. Yufei Song et al. reported Sr_0.9_Ce_0.1_Fe_0.8_Ni_0.2_O_3-δ_ nanocomposites for solid oxide fuel cell (SOFC) cathodes, resulting in higher ORR activity, low conductivity, good stability, and reduced thermal expansion coefficient (Fig. 4a) [135]. The nanocomposite was synthesized by the exsolution method with fine temperature control in a bulb, and the nanocomposite consists of a single perovskite main phase, a Ruddlesden-Popper (RP) second phase, and surface-decorated NiO and CeO_2_ minor phases. These components are intimately mixed in the nanodomain, with the NiO and CeO_2_ phases mainly located on the surface of the main phases. In this nanocomposite, the RP phase enhanced the oxygen bulk diffusion and the resulting NiO and CeO_2_ nanoparticles promoted the oxygen surface process, facilitating the surface to the main phase and oxygen migration. Also, spray pyrolysis is used to produce particulate nanocomposites, respectively, providing control over the morphology and phase distribution of the composite. The ratio of the two substances in the particles can be precisely controlled by adjusting the ratio of the precursor, among other factors. The particle size can be controlled by varying the amount of precursor, which in turn affects the crystallinity. Also, the temperature can be used to regulate the particle size, with the amount of carrier gas according to the residence time. A feeder is designed to control the amount of precursor supplied and the size of the initially injected precursor. Javier Zamudio-García et al. prepared La_0.98_Cr_0.75_Mn_0.25_O_3-δ_-Ce_0.9_Gd_0.1_O_1.95_ (LCM-CGO) nanocomposite layers with different LCM contents, between 40 and 60 wt%, to utilize the benefits of the LCM such as high redox stability and the benefits of the CGO such as high ionic conductivity (Fig. 4c) [62]. They show compatibility with LaCrO_3_-based electrodes and high ionic conductivity thanks to limited grain growth, resulting in maintaining nanoscale microstructures even after annealing at 1000 ℃. Their synergistic effect results in efficient and durable symmetrical electrodes with high resistance and high efficiency. In addition, the shoulder-to-shoulder structure is also obtained by mixing different precursor solutions in one solution to obtain a perovskite nanocomposite simultaneously. Lujian Jia et al. developed a dual-phase membrane Ce_0.9_Pr_0.1_O_2-δ_-Pr_0.1_Sr_0.9_Mg_0.1_Ti_0.9_O_3-δ_ (CPO-PSM-Ti) with good chemical stability and mixed oxygen ion–electron conductivity in a reducing atmosphere for H_2_ purification by sol–gel method and high-temperature sintering (Fig. 4b) [136]. The composites with mixed conductivity and good stability show limited electronic conductivity and excellent chemical stability thanks to Ce_0.9_Pr_0.1_O_2-δ_ (CPO).

Surface decoration can be synthesized by gas-based deposition techniques (e.g., physical vapor deposition) or liquid-based approaches (e.g., impregnation and co-precipitation). Assuming AO exhibits greater reducibility compared to BO, the variance in oxygen chemical potential between the atmosphere and the solid surface triggers an oxygen flux, prompting the formation of reduced A at or just beneath the surface. In some situations, this method can also be considered an "in-situ separation process" because the metal/oxide catalyst platform is automatically generated under the working conditions of the catalytic reaction [137–139].

## Nanocomposite-based applications

### Fuel cells

**Fig. 5:**
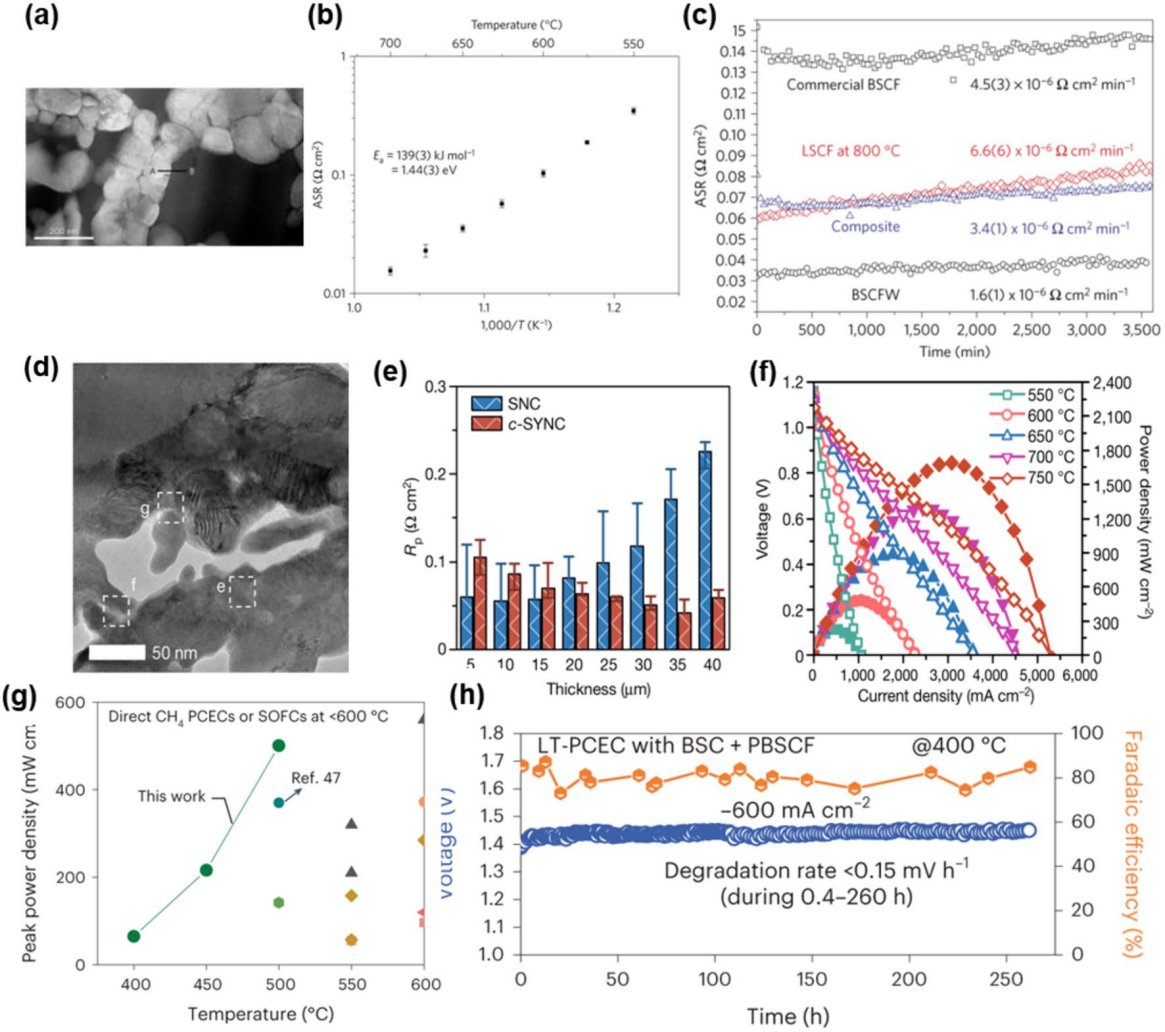
Perovskite nanocomposite applications for Fuel cell **a**. Dark-field image of BSCFW. Area specific resistance of BSCFW. **b**. the various temperature and **c**. various samples [53]. **d**. HRTEM, **e**. area specific resistance of the Sr_x_(Y_y_(Nb_0.1_Co_0.9_)_1–y_)O_3−δ_ composite, **f**. voltage and power density versus current density curves of an anode-supported H_2_/air SOFC with Sr_x_(Y_y_(Nb_0.1_Co_0.9_)_1–y_)O_3−δ_ composite [145]. **g**. Comparison of electrochemical full-cell performance on methane. **h**. Long-term stability test at 400 ℃ with a charging current density of 600 mA/cm^2^ [146]

Fuel cells utilizing hydrogen gas as their primary source are widely recognized as a promising energy storage solution thanks to their ultra-high calorific value of hydrogen (282 kJ/mol), cost-effectiveness, and high efficiency [140, 141]. For efficient fuel cell systems, oxides-perovskite nanocomposites have attracted significant attention as outstanding materials thanks to the following reasons: (1) Structural variations in oxide perovskites (i.e., octahedral distortion, B-cation displacement, and octahedral tilting) derive various polymorphs [142, 143], (2) the majority (~ 90%) of metal elements in the periodic table can be stabilized within the perovskite framework [144], and (3) nanocomposite structures enable high thermal, electrical, and mechanical stabilities. Figure 5a shows the dark-field TEM image of a 0.5Sr_0.5_(Co_0.7_Fe_0.3_)0.6875W_0.3125_O_3−δ_ (BSCFW) anode, which is a self-assembled composite prepared through simple solid-state synthesis, consisting of B-site cation ordered double perovskite and disordered single perovskite oxide phases [53]. BSCFWs, of themselves, assemble into composite structures, which prevents loss of external surface through agglomeration and limits formation of oxygen vacancies at operating temperatures. Thanks to their unique phase transition, low area specific resistance with chemical and mechanical stability was observed at the temperature range around 500–700 ℃, quasi-epitaxial interfaces between the phases (Fig. 5b,c).

Despite their advantages, thermo-mechanical instability presents a significant bottleneck, resulting in a substantial internal strain gradient. This issue stems from thermal expansion mismatches among various fuel cell components, leading to concerns such as cell degradation, delamination, and eventual failure. To circumvent the limitations, Y. Zhang et al. developed reactive sintering to combine a cobalt-based perovskite with high electrochemical activity and large thermal-expansion coefficient with a negative-thermal-expansion material (Fig. 5d). Thereafter, they form composite electrodes that do not have an issue regarding thermal-expansion mismatch with the electrolyte [145]. The Sr_x_(Y_y_(Nb_0.1_Co_0.9_)_1–y_)O_3−δ_ composite electrode demonstrates an area-specific ohmic resistance value of 0.041 Ω·cm^2^ for a thickness of 35 μm at 600 ℃ (Fig. 5e). The peak power density from an SOFC button cell employing the c-SYNC cathode reaches 1690 mW/cm^2^ at 750 °C (Fig. 5f).

However, the persistently high operating temperature (exceeding 500 ℃) remains a hurdle to enhancing both power output and stability. This challenge arises from issues like rapid corrosion of electrolytes and cell detachment. Recently, F. Liu et al. report a simple and scalable approach for fabricating ultrathin, chemically homogeneous, and robust proton-conducting electrolytes. They demonstrate an in-situ formed composite electrode, Ba_0.62_Sr_0.38_CoO_3−δ_–Pr_1.44_Ba_0.11_Sr_0.45_Co_1.32_Fe_0.68_O_6−δ_, which significantly reduces ohmic resistance, electrode–electrolyte contact resistance and electrode polarization resistance [146]. As shown in Fig. 5g and h, the PCECs attain high power densities in fuel-cell mode (~ 0.75 W/cm^2^ at 450 ℃ and ~ 0.10 W/cm^2^ at 275 ℃) and exceptional current densities in steam electrolysis mode (−1.28 A/cm^2^ at 1.4 V and 450 ℃). The current research focus underscores the promising potential of perovskite nanocomposites in fuel cells, highlighting their structural resilience and electrochemical prowess. Continued progress in materials development and design is essential to further improve fuel cell efficiency and reliability for real-world applications.

### Electrochemical water splitting

**Fig. 6:**
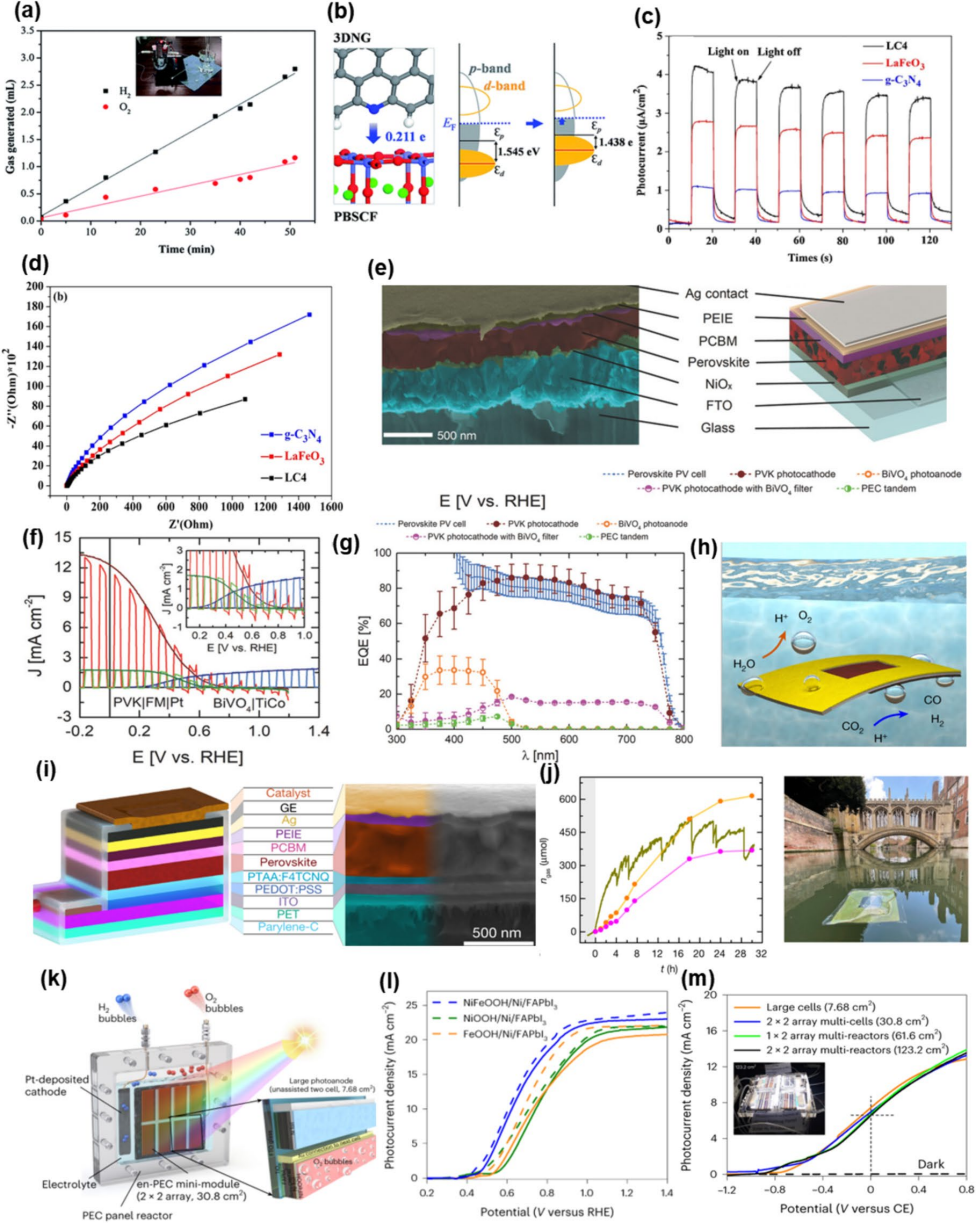
Electrochemical water splitting systems with perovskite nanocomposites. **a**. The charge transfer from 3DNG to PBSCF. **b**. and the schematic band diagrams of PBSCF and PBSCF with 3DNG [152]. **C**. Transient photocurrent responses and **d**. Nyquist plots of the as-synthesized samples under intermittent visible light irradiation [153]. **e**. SEM (left), schematic images (right),** f**. current density, and **g**. EQE of the PEC water splitting tandem cell [156]. **h**. Architecture of a wired perovskite photocathode,** i**. SEM image of the leaf-like PEC device, and **j**. Outdoor test on the River Cam (UK), in front of the Bridge of Sighs, St John’s College [157]. **k**. An all-PSK-based en-PEC system for large-scale, unassisted solar water splitting was constructed by connecting in parallel 2 × 2 arrays of enlarged NiFeOOH-Ni-FAPbI_3_ photoanodes, **l**. Current density of the samples in 1 M KOH electrolyte, and **m**. current densities of the unassisted large cell photoanodes show the effects of different active cell areas on their PEC performance [155]

Electrochemical water splitting (2H_2_O → 2H_2_ + O_2_) cells has been widely studied because of their advantages of solar energy applicability with low external bias, abundant water resources, simple equipment, green synthesis process, and high yield [147–149]. For the efficient electrochemical water splitting system, electrocatalysts in the anode and cathode are crucial to generate hydrogen gases and react peroxide ions. Perovskite structures have attracted attention because their conduction-band minimum and the valance band maximum straddle the water redox potentials, facilitating an efficient water splitting reaction [151]. Many of oxide perovskite nanocomposites have been studied by adapting conductive 2D layers to further improve their PEC water splitting performance. Y. Bu et al. reported cation-ordered perovskite (PrBa_0.5_Sr_0.5_)_0.95_Co_1.5_Fe_0.5_O_5+δ_ nitrogen-doped graphene (3DNG) nanocomposites for hydrogen generation electrodes (Fig. 6a,b) [152]. The large amount of hydrogen is evolved up to 0.859 μL/s because of acceleration of charge separation in 3DNG, confirmed by density functional theory (DFT) calculation in Fig. 6b. Y. Lu et al. implemented La_1-x_Sr_x_CoO_3_-Ti_3_C_2_T_x_ MXene-Ni electrodes high current density over 10 mA/cm^2^ with low overpotential of 279 mV (Fig. 6c,d) [153]. Recently, some researchers discovered that active polymers with 2D materials accelerate charge separation, improving water splitting performance of nanocomposites [154]. Figure 6c shows a chronoamperometric curve of the LaFeO_3_-g-CN formed by a facile quasi-polymeric calcination method. The current density of the nanocomposite was measured to be 4 μA/cm^2^ at 0 V vs RHE. This improved current density is attributed to the decrease in charge transport resistance due to the impedance matching, evaluated by Nyquist plot (Fig. 6d).

Despite such advantages, they have inherent limitations to produce large amounts of hydrogen owing to their large band gap over 3 eV, low photoconversion efficiency, and low charge density. Therefore, many researchers have tried to implement high hydrogen evolution reactions using halide perovskite nanocomposites with a photovoltaic-electrochemical (PV-EC) water splitting approach. Figure 6e shows the electrochemical full cell with MAPbI_3_-Polyethylenimine (PEIE)-thin phenyl C_61_ butyric acid methyl ester (PCBM) composites with Pt catalysts [156]. Their current density was measured to be 12 mA/cm^2^ at 0 V vs RHE, which implies the high applied-bias photon-to-current efficiency (ABPE) (Fig. 6f). This result is attributed to high external quantum efficiency (EQE) of the MaPbI_3_ perovskite layers across the visible wavelength region (Fig. 6g). Moreover, the leaf-like PEC devices with unassisted and floating halide perovskite nanocomposite were designed for highly efficient HER performance (Fig. 6h,i) [157]. The halide perovskite photocathodes deposited onto indium tin oxide-coated polyethylene terephthalate achieved an activity of 4266 µmol/gh using platinum catalysts (Fig. 6j). In recent studies, band structure engineering in all-perovskite nanocomposite cells have emerged as a focus area. The aim is to produce significant quantities of hydrogen gas by optimizing band alignment for superior carrier extraction performance. Figure 6k shows the schematic illustration of the formamidinium lead triiodide (FAPbI_3_) perovskite-based photoanodes encapsulated by an Ni foil-NiFeOOH electrocatalyst [155]. The current density and maximum ABPE of the NiFeOOH-Ni-FAPbI_3_ photoanode were measured to be 22.82 mA/cm^2^ at 1.23 V vs RHE and 7.24% in Fig. 6l. To achieve high hydrogen evolution reaction (HER) performance, NiFeOOH-Ni-FAPbI_3_ photoanodes were successfully upscaled from 0.25 to 123.2 cm^2^ (500 times larger) with minimal decrease in solar-to-hydrogen (STH) efficiency (less than 15%). This was accomplished by enlarging the unit cell size, adopting a multi-cell approach, and employing a multi-reactor approach (Fig. 6m).

### Electrochemical CO_2_ reduction

**Fig. 7:**
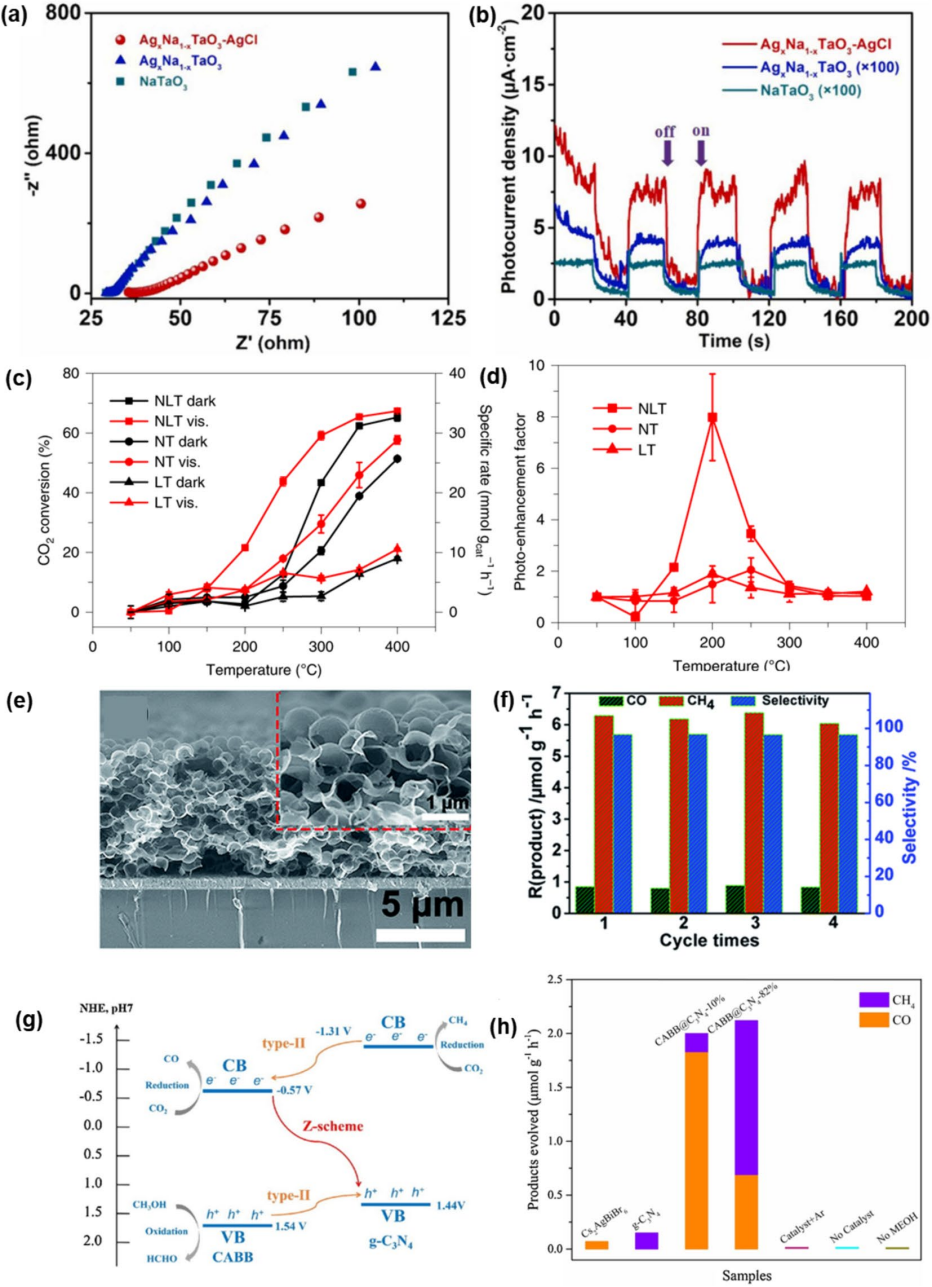
Electrochemical CO_2_ reduction system with perovskite nanocomposites. **a**. EIS Nyquist plots and **b**. photocurrent density of Ag_x_Na_1_ − _x_TaO_3_-AgCl, Ag_x_Na_1_ − _x_TaO_3_ and NaTaO_3_ [158]. **c**. CO_2_ conversion and **d**. photo-enhancement factor under visible light over the temperature range of 50–400 ℃ by NiO_*x*_-La_2_O_3_-TiO_2_ (NLT), NiO_*x*_-TiO_2_ (NT) and La_2_O_3_-TiO_2_ (LT) catalysts [159]. **e**. Cross-sectional SEM image of MRGO (3D macro porous RGO structure). **f**. Recycling tests of CsPbBr_3_ NC-BZNW-MRGO for 4 times [162]. **g**. Energy level diagrams and charge transfer routes in CABB-C_3_N_4_-10% Type II (orange arrows) and CABB-C_3_N_4_-82% Z-scheme heterojunction (red arrow) **h**. Photocatalytic CO_2_ reduction performance of g-C_3_N_4_, Cs_2_AgBiBr_6_, Cs_2_AgBiBr_6_-C_3_N_4_-10% and Cs_2_AgBiBr_6_-C_3_N_4_-82% [163]

Oxide perovskite-based nanocomposites for CO_2_ reduction are paired with semiconductors capable of absorbing the visible wavelength region. This combination enhances photoconversion efficiency. For example, Guo et al. synthesized a highly-crystalline Ag_x_Na_1-x_TaO_3_–AgCl heterojunction by a one-step flux method, where CB and VB of Ag_x_Na_1-x_TaO_3_ were positioned between those of AgCl [158]. From EIS Nyquist plots and photocurrent density of NaTaO_3_, Ag_x_Na_1-x_TaO_3_, and Ag_x_Na_1-x_TaO_3_–AgCl photocatalysts (Fig. 7a,b), the crystalline Ag_x_Na_1-x_TaO_3_–AgCl heterojunction improved migration and separation of photogenerated charges. With the efficient band alignment approach, surface adsorbates also play a critical role in photo-assisted CO_2_ activation. T. H. Tan et al. developed NiO_x_-La_2_O_3_-TiO_2_ nanocomposites, where the La_2_O_3_-TiO_2_ facilities adsorption of CO_2_, which contributes to sustained HCO_2_* formation and conversion [159]. Notably, the performance delivered by NiO_x_-La_2_O_3_-TiO_2_ at 250 ℃ under illumination (21.9 /g_cat_, 43.8% conversion) was comparable to the catalyst activity at 300 ℃ without illumination (21.7 /g_cat_, 43.7% conversion), representing an effective 50 ℃ decrease in the temperature requirement (Fig. 7c and 7d).

Halide perovskite nanocomposites have also gained prominence as excellent CO_2_ reduction materials due to their CB levels being more negative than the reduction potential of CO_2_, high molar extinction coefficient, and low exciton binding energy [160, 161]. Figure 7e shows the CsPbBr_3_-branched ZnO nanowire (BZNW)/microporous r-GO nanocomposite, where the nanocomposites have Type II band alignment for high carrier extraction efficiency [162]. Owing to the efficient band structure and low charge transfer resistance, a boosted CO_2_ reduction performance was achieved with a photoelectron consumption rate of 52.02 µmol/g_cat_h under visible light irradiation with desirable CH_4_ productivity of up to 96.7% (Fig. 7f). More recently, new Type II heterojunction with a Z-scheme charge-transfer model was proposed [163]. In a Z-scheme heterojunction, photogenerated electrons with a lower CB position would recombine with holes with a higher VB position, leaving electrons and holes at the CB and VB, respectively. Figure 7g shows the schematic illustration of the band structure of Z-scheme heterostructures of Cs_2_AgBiBr_6_-g-C_3_N_4_ Z composites. The nanocomposites exhibit improved CO_2_ reduction performance, giving the production rate of above 2.0 μmol/gh without high chain hydrocarbons products or hydrogens (Fig. 7h).

### Supercapacitor

**Fig. 8:**
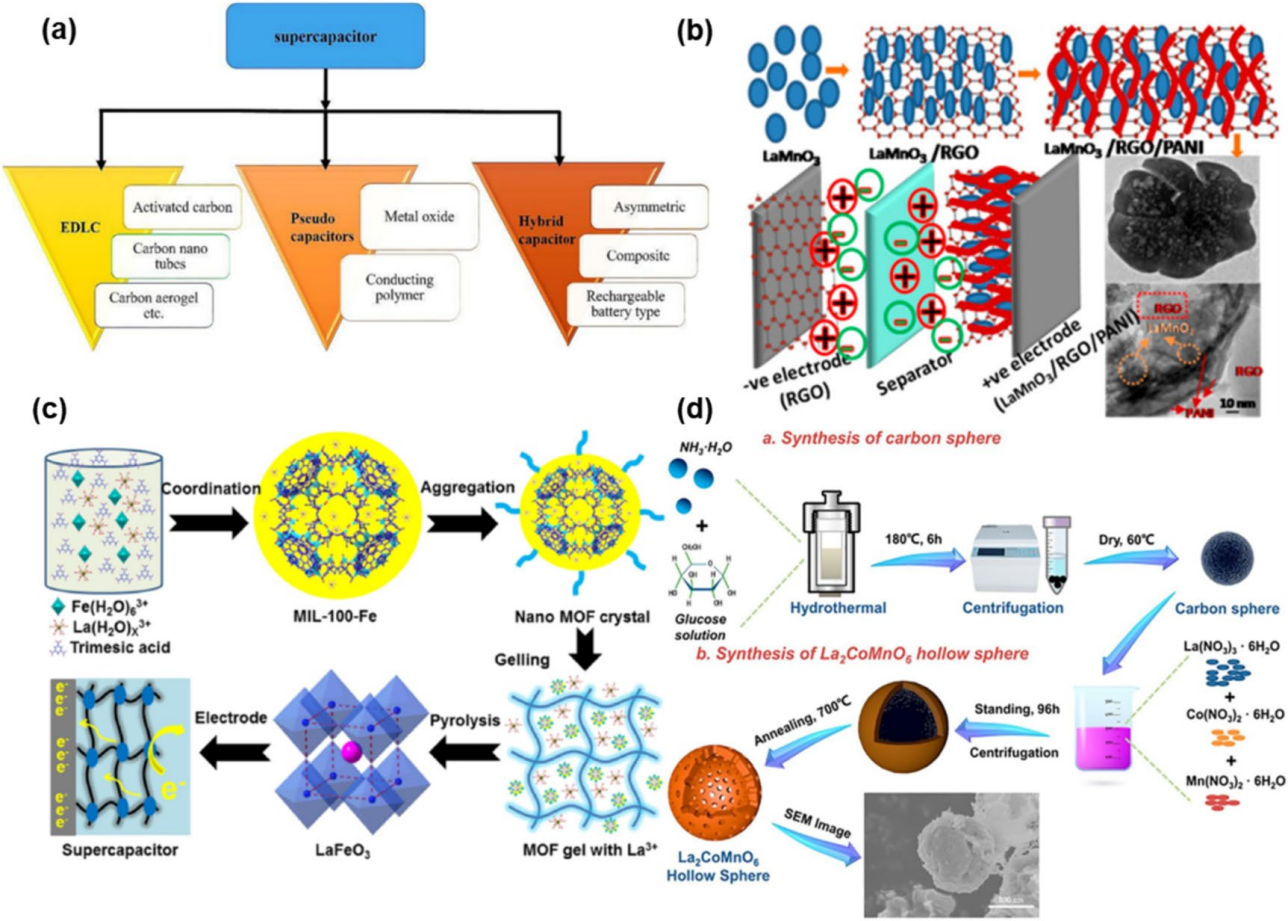
Supercapacitors with perovskite nanocomposites. **a**. Classification of supercapacitors [194]. **b**. Incorporation of RGO, PANI, and LaMNO_3_ [168]. **c**. Fabrication method of mesoporous LaFeO_3_ [169]. **d**. Fabrication method of hollow sphere La_2_CoMnO_6_ [170]

The supercapacitor (SC) plays a crucial role in energy conversion and storage systems due to its exceptional attributes: high-power density, ultrafast charge–discharge rates, and extended cycle life. It serves as a vital link between traditional capacitors and rechargeable batteries. Perovskite nanocomposites exhibit great promise owing to their adjustable electrical and ionic conductivity and substantial charge storage capacity (Fig. 8a). Moreover, easy modulation of oxygen vacancies in perovskite materials provides high energy storage capacity and electrical stability. For example, Co-based perovskites, known for their advantages such as ion transportation of oxygen, high conductivity, outstanding electrocatalytic activity, are used for enhancing energy density by partial substitution of cation B sites in ABX_3_ perovskite structures [164]. Therefore, increasing oxidation states of cation B sites or concentration of oxygen vacancies can be achieved through partial substitution of Fe atoms in Co-based perovskite, thereby achieving both high energy density and stability. As a result, optimal composition, SrFe_x_Co_1-x_O_3-y_ (SCF-x), derived from SrCoO_3_-SrFeO_3_, shows a noteworthy energy density of 194.85 Wh/kg and corresponding power density of 1798.61 W/kg. Also, involving graphene oxide (GO) materials into perovskites proposes another way to improve the performance of SC, thanks to low interface impedances [165–167]. For instance, the incorporation of reduced GO (RGO) and conductive polyaniline with LaMnO_3_ significantly enhanced the energy density (25 Wh/kg) at the power density of 18 kW/kg and stability [168]. Moreover, a composite of RGO and LaAlO_3_ exhibited a specific capacitance of 111 F/g at a current density of 2.5 A/g, outperforming the 100 F/g observed for LaAlO_3_ alone (Fig. 8b).

Furthermore, the formation of nanocomposites has been explored to achieve high surface area. For instance, mesoporous LaFeO_3_ combined with an metal-organic framework (MOF) has demonstrated outstanding performance (Fig. 8c) [169]. The uniform and high porosity of the mesoporous structure reduce ion diffusion resistance, facilitating rapid charge transfer. Additionally, the large surface area of the mesoporous structure contributes to minimizing electrode polarization. Consequently, supercapacitors (SCs) incorporating mesoporous LaFeO_3_ exhibit an energy density of 34 Wh/kg at a power density of 900 W/kg, with 92.2% retention after 5000 cycles. Another strategy to enhance the surface-to-volume ratio involves utilizing template impregnation. This method shapes perovskites into useful forms to increase surface area. For instance, hollow sphere La_2_CoMnO_6_ (HS-LCMO) fabricated by this method has shown promise for SCs (Fig. 8d). This hollow spherical structure significantly boosts a surface-to-volume ratio, thereby reducing transport length of both mass and charge transport. Therefore, the expanded surface area provides additional reactive sites, leading to increase in specific capacity. Also, abundant mesopores promote transmission of electrons while the hollow structure improves rapid charge–discharge process. Based on these benefits, HS-LCMO shows a noticeable energy density of 65.8 Wh/kg at a power density of 1000 W/kg [170].

### Optoelectronics

**Fig. 9:**
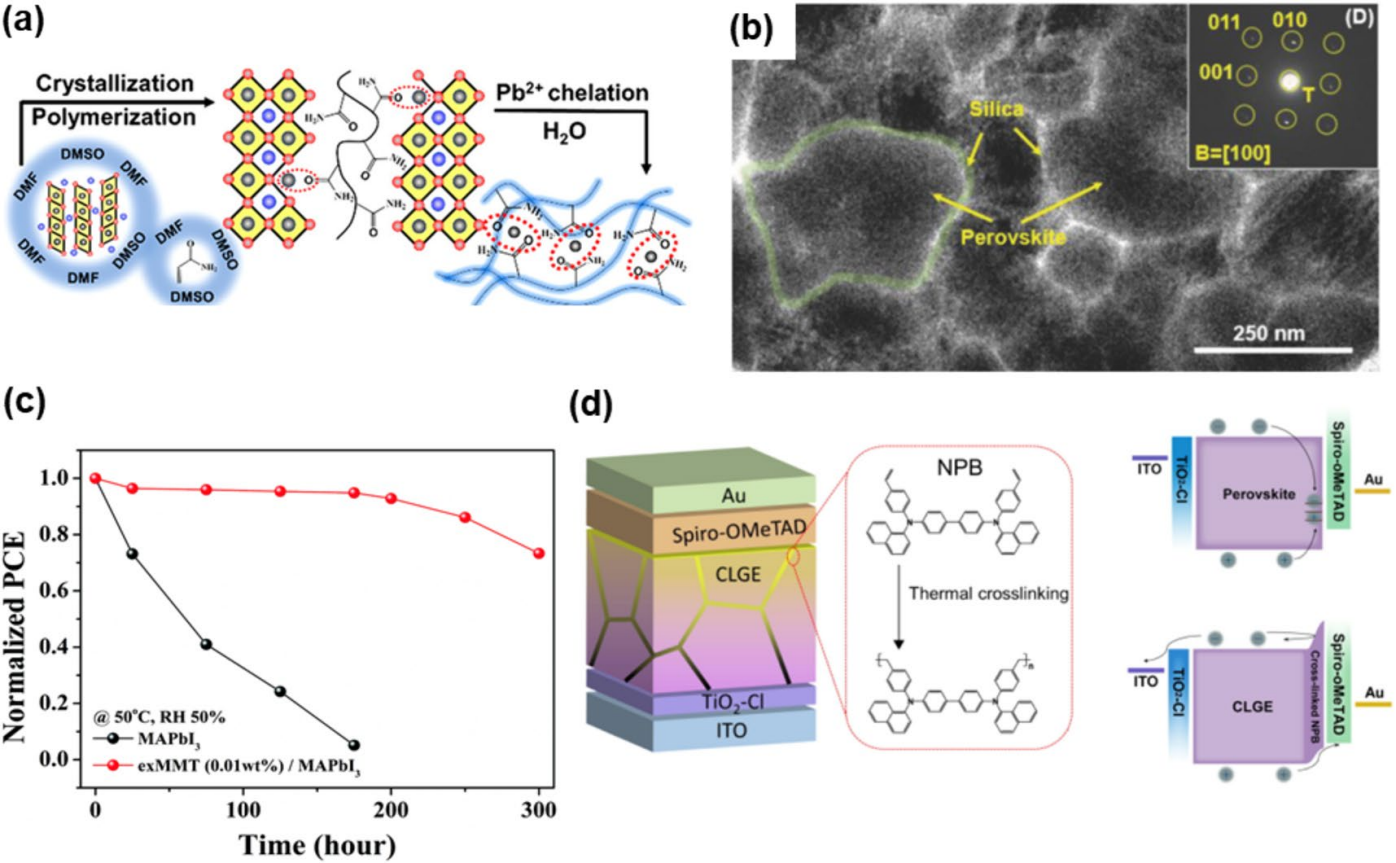
PSCs with perovskite nanocomposites. **a**. Lead-leakage blocking strategy and fabrication process with schematic illustration of additive-to-polymer transformation in solution, solid, and water [175]. **b**. Bring-field TEM image showing FAPI-T film consists of silica-encapsulated perovskite grains [178]. **c**. Damp-heat tests of PSCs. Normalized PCEs of the PSCs with pristine MAPbI_3_ and exMMT (0.01 wt%)-MAPbI_3_ as a function of storage time (50 ℃, RH 50%) [174]. **d**. Schematic device configuration with CLGE for perovskite solar cell. Cross-linking of NPB molecules occurs via the polymerization between styrene groups following a thermal treatment [177]

Halide perovskite lattices typically exhibit high distortion, weak interatomic bonding, and high density of local defects. These intrinsic qualities attest to the volatile layer susceptible to thermal and illumination-induced degradation and decomposition as well as ion migration. Thus, involving nanocomposites to mitigate the breakdown of the active layer strengthens the lattices, leading improvement in its morphology, crystallinity, and chemical stability [171–173]. Also, Table 1 demonstrates the improvement in the functionality of optoelectronic devices resulting from the application of perovskite nanocomposite. For example, Niu et al. performed in-situ polymerization of acrylamide (AAm) monomers within the perovskite layer (CS_0.05_ (FA_0.90_MA_0.10_)_0.95_Pb(I_0.90_Br_0.10_)_3_) of an inverted PSC (Fig. 9a) [175]. The resulting lead-chelating polymer network not only passivates the defects of perovskite, thereby achieving a PCE of 22.1%, but also prevents the dissolution of lead ions in water, holding up to 94% rejection rate upon directly immersing the unencapsulated devices into water. Huang et al. achieved half-year stable PSC by employing a montmorillonite (MMT)-CH_3_NH_3_PbI_3_ nanocomposite. The 1 nm-thick MMT formed a protective shell outside of the perovskite crystals, slowing the aging effect of light, heat and humidity without sacrificing the PCE (Fig. 9c) [174]. On the other hand, while lead-containing perovskite still proves the most promising for optoelectronics applications due to its relatively higher stability compared to other group IV elements, the adverse health and environmental impacts of lead leakage remain significant concerns. This has spurred research into Pb-encapsulation methods to mitigate these effects. Lead leakage is predominantly triggered by exposure to moisture, a vector for degradation that not only affects the layer interfaces but is also directly proportional to the perovskite grain size [176]. Encapsulation strategies that target the grain boundaries have demonstrated effectiveness in extending the operational longevity of PSCs. To list a few, Xiao et al. developed NPB-Cs_0.05_FA_0.9_MA_0.05_PbI_2.85_Br_0.15_ nanocomposites by introducing the cross-linkable p-type semiconducting molecules NPB into the anti-solvent (Fig. 9d) [177]. A compact and conductive layer is thus formed at perovskite grain boundaries, facilitating both hole extraction and device stability (Fig. 9b) [178]. Liu et al., enhanced the stability and efficiency of FA-based lead iodide perovskite solar cells to nearly 20% efficiency with 97% retention after 1000 h in ambient conditions by integrating a tetraethyl orthosilicate (TEOS) hydrolysis process. This process encapsulates perovskite grains with in-situ formed amorphous silica layers (SiO_2_-FA_0.85_Cs_0.15_PbI_3_).

**Table 1:** Improved performance or reliability in optoelectronic applications with nanocomposite structures

	Compound	Structure	Strategies	Synthesis	PL Peak (nm)	FWHM (nm)	PLQY (%)	PL lifetime (ns)	Photo stability	EQE (%)
[133]	FAPbBr_3_/CsPbBr_3_	Core/shell	In‑situ synthesis	Heat‑up	504	3	82 → 93	30.2 → 40.1	A: ~ 60% (20 w) B: ~ 10% (20 w)	1.03 → 8.1
[196]	CsPbI_3_/KI	Core/shell	Post‑synthesis	Hot‑injection	640	31	75 → 96	7 → 10	~ 50% (10 h) ~ 50% (1.6 h)	16 → 21.3
[78]	CsPbBr_3_/Cs_4_PbBr_6_	Core/shell	In‑situ synthesis	Hot‑injection	516	NA	84.4 → 96.2	16.2 → 9.1	A: ~ 52.5% (7 d) B: < 10% (2 d)	NA
[39]	MAPbBr_3_/(OA)_2_PbBr_4_	Core/shell	In‑situ synthesis	LARP	520	25	NA → 88	NA	A:88.12 → 55.46 (80 d)	NA
[198]	MAPbBr_3_/SiO_2_/PVDF	Core/shell	Post‑synthesis	Capillary forces	530	27	NA → 85.5		NA	NA
[199]	CsPbBr3/SiO2/Al2O3	Core/shell	Post‑synthesis	Sol–gel	519	25	67 → 90	11.2 → 20.8	A:100‑ > 90% (300 h) B:100‑ > 10% (72 h)	NA
[80]	CsPbBr_3_/MPMs/SiO_2_	Core/shell	Post‑synthesis	Hydrolysis‑encapsu‑ lation	518	23	93 → 84	14.36 → 29.59	A: ~ 48% (30 d) B: < 10% (5 h)	
[197]	CsPbBr_3_/LP/SiO_2_	Core/shell	Post‑synthesis	Hot‑injection	NA	19	44 → 90.5	9 → 15	A: ~ same (168 h) B: ~ 50% (168 h)	NA
[193]	FAPbBr_3_/CsPbBr_3_	Core/shell	Post‑synthesis	LARP	513	20	NA	NA	A: ~ 90% (1200 min) B: ~ 60% (1200 min)	NA
[195]	CsPbBr_3_‑MABr	Quasi core/shell	Post‑synthesis	spin coating	525	20	NA → 80	NA	A: ~ 50% (10.42 min) B: ~ 50% (7.65 min)	17 → 20.3
[150]	MAPbBr_3_:PBD	Nanoplatelet	Post‑synthesis	LARP	529	20	65 → 85	100 → 2.7 µs	NA	0.038 → 0.48

**Fig. 10:**
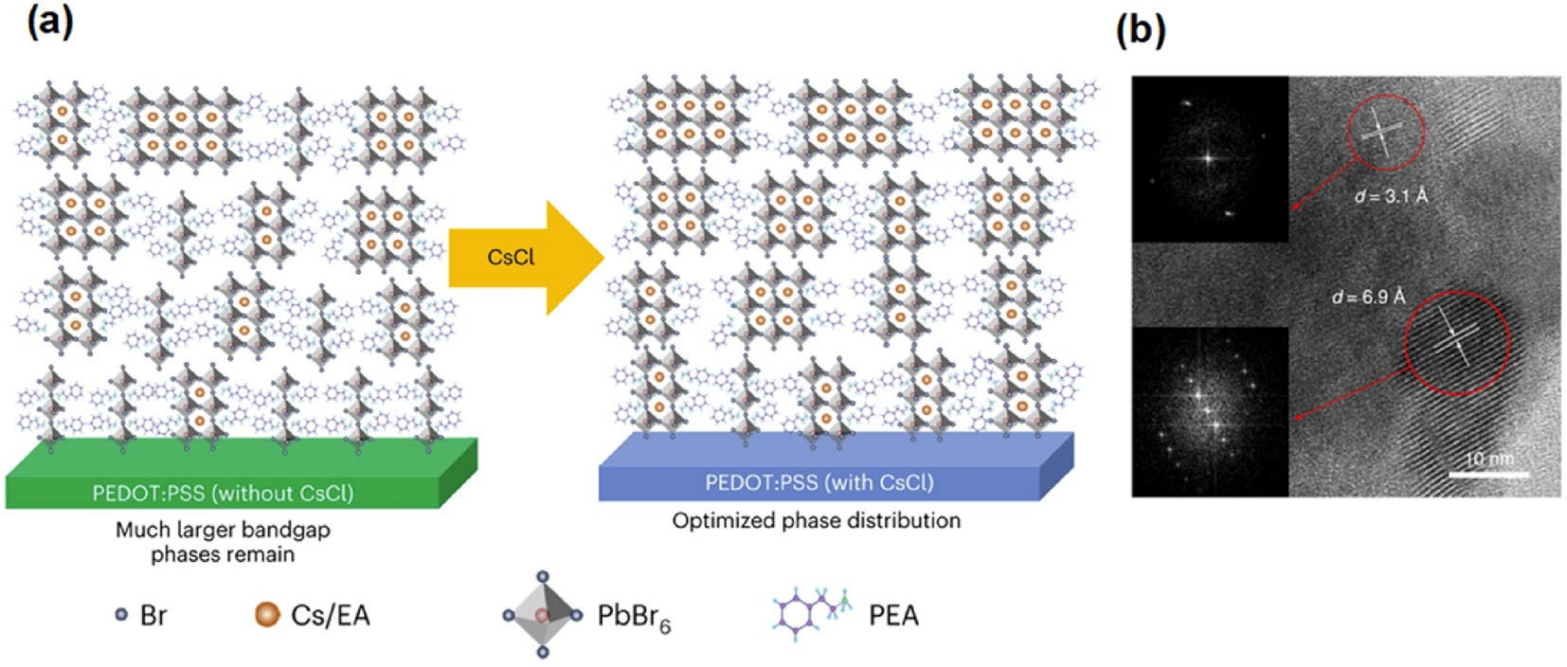
PeLEDs with perovskite nanocomposites. **a**. Proposed schematic of the rearrangement of the phase distribution of quasi-2D perovskites after CsCl diffusion [180]. **b**. HR-TEM image of a PPBH sample. Insets, Fast Fourier transforms of the quasi-2D/3D crystalline regions [181]

The incorporation of a mixed-phase perovskite layer has introduced a novel nanocomposite into perovskite-based light emitting diode (PLED) devices. Quasi-2D perovskites, or low dimension perovskites, introduce self-assembled quantum wells with its larger electron binding energy. The mixed-phase characteristics (as the formation energies for phases with different low dimensions, *n*, are close) of quasi-2D perovskites allow photocarriers to transfer rapidly and efficiently from higher to lower bandgap regions, facilitating to an accumulation of carriers in the recombination centers and significantly advancing photo luminance [179]. Thus, precise adjustment of the phase distribution within the quasi-2D perovskite composite has been identified as crucial for improving device performance. Chu et al.’s reported the growth of perovskite films atop substrates containing caesium-chloride, which avoids the predominance of 2D layers with large bandgaps (n = 1) and ameliorating associated inefficiencies (Fig. 10a) [180]. In addition, mixed-dimensional, or 2D/3D perovskite heterostructures has aimed to harness the stability of 2D phases and charge transport capability of 3D phases (Fig. 10b). Zhao et al. integrate a composite of quasi-2D and 3D perovskites with a poly-HEMA capping layer [181]. Photogenerated excitations rapidly migrate within the quasi-2D phase, and undergoes radiative recombination in the 3D regions, while the polymer layer is critical in preventing luminescence quenching, contributing to an EQE of 20.1%. An extensive review on quasi-2D PeLED has been conducted by Zhang et al., where photoelectronic properties and specific device engineering strategies are covered [179].

## Outlooks

In this review, we have summarized recent advances of perovskite nanocomposites on formation processes as their structures, as well as their recent progress in electrochemical and optoelectronic applications. Despite the potential of pure perovskite materials, they have been plagued by instability and low efficiency. In this regard, nanocomposite structures offer a promising solution to overcome the intrinsic shortcomings of perovskites, including stability issues, energy storage performance, power conversion efficiency (PCE), and high recombination rates. Their impressive development signifies their potential as a promising candidate for various applications. In light of these advancements, it seems prudent to identify some of the areas that remain under-researched.

First, despite the numerous research attempts that have been made, the most significant issue is stability. A multitude of environmental factors, including humidity, oxygen, temperature, and UV light, can affect the stability of perovskite composites. Furthermore, it is currently unclear whether the nanocomposite structure is capable of completely preventing oxygen and moisture from accessing the NCs over an extended period of time. Ultimately, the goal is to optimize stability factors, such as structures, composition, and morphology of perovskite nanocomposites. This will result in the production of core/shell nanostructures that are completely covered by a robust inorganic shell material, such as metal chalcogenides, oxides, or fluorides.

Second, we need to further improve size variation and uniformity of nanocomposite formation. For example, sol–gel and solid-phase synthesis are commonly used for in-situ synthesis, but result in crystal sizes often on the order of hundreds of nanometers and non-uniform distribution. In addition, due to the different arrangement of atoms on each surface of perovskites, shells grow preferentially on a certain surface, resulting in uneven shell thickness and increased lattice strain. This can affect the properties of the core/shell structures, so novel approaches such as spray pyrolysis and surface decoration have been tried to achieve uniform distribution and fine size control. We need further development in advanced synthesis, leading to precise size control with minimal variation.

Third, there is still room to further improve the performance of perovskite nanocomposites. For instance, oxide perovskite nanocomposites often exhibit low conductivity and low carrier densities at interfaces, leading to poor electrochemical reactions. The small number of carriers formed and the low carrier extraction efficiency due to the wide band gap further contribute to suboptimal electrochemical performance. Therefore, research on materials and structures that induce higher carrier densities and ensure high crystallinity at the surface of oxide perovskite nanocomposites is essential. Additionally, optimizing the band alignment of perovskite nanocomposites is expected to enhance performance further. Future research should focus on developing novel structures that can be integrated with conventional halide perovskites to address their intrinsic instability, while simultaneously achieving efficient charge separation, increased catalytic active sites, and other favorable outcomes. The synthesis of nanocomposites involves the formation of new lattice structures and inter-material synergies. Thus, it is possible to control different nanocomposite structures and predict or explain their performance using Density Functional Theory (DFT) and machine learning.

The versatility of perovskite nanocomposites may extend to their effective integration with various 2D materials and nanostructures, such as the transition metal (TM) dichalcogenides, TM carbides, MXenes and TM nitrides. This integration holds the promise of unveiling novel functionalities and exploring physical phenomena that were unveiled. Targeted nanocomposites can be fabricated by combining multiple active components for specific applications. In addition to synergistic effects, the infinite number of interfaces and flexible configurations with strong interactions will be a key factor in improving catalytic performance. Thus, the intrinsic advantages of the perovskite nanocomposites, including the convenience of combining various materials, low process cost, and compatibility with various materials, will bring a new world in the electrochemical and optoelectronic era with new physics and new applications.

## Data Availability

The review is based on the published data and sources of data upon which conclusions have been drawn can be found in the reference list.
